# PHIRI: lessons for an extensive reuse of sensitive data in federated health research

**DOI:** 10.1093/eurpub/ckae036

**Published:** 2024-07-01

**Authors:** Juan González-García, Javier González-Galindo, Francisco Estupiñán-Romero, Martin Thißen, Ronan A Lyons, Carlos Telleria-Orriols, Enrique Bernal-Delgado, Population Health Information Research Infrastructure, Petronille Bogaert, Nienke Schutte, Pascal Derycke, Sarah Aldridge, Andrea Schmidt, Lorenz Dolanski-Aghamanoukjan, Jennifer Zeitlin, Marianne Philibert, Hanna Tolonen, Mikka Gissler, Carmen Rodríguez-Blázquez

**Affiliations:** Data Sciences for Health Services and Policy Research, Institute for Health Sciences in Aragón (IACS), Zaragoza, Spain; Data Sciences for Health Services and Policy Research, Institute for Health Sciences in Aragón (IACS), Zaragoza, Spain; Data Sciences for Health Services and Policy Research, Institute for Health Sciences in Aragón (IACS), Zaragoza, Spain; Department of Epidemiology and Health Monitoring, Robert Koch Institute, Berlin, Germany; Population Data Science, Swansea University Medical School, Faculty of Medicine, Health, and Life Science, Swansea University, Swansea, Swansea, UK; Data Sciences for Health Services and Policy Research, Institute for Health Sciences in Aragón (IACS), Zaragoza, Spain; Data Sciences for Health Services and Policy Research, Institute for Health Sciences in Aragón (IACS), Zaragoza, Spain

## Abstract

**Background:**

The extensive and continuous reuse of sensitive health data could enhance the role of population health research on public decisions. This paper describes the design principles and the different building blocks that have supported the implementation and deployment of Population Health Information Research Infrastructure (PHIRI), the strengths and challenges of the approach and some future developments.

**Methods:**

The design and implementation of PHIRI have been developed upon: (i) the data visiting principle—data does not move but code moves; (ii) the orchestration of the research question throughout a workflow that ensured legal, organizational, semantic and technological interoperability and (iii) a ‘master–worker’ federated computational architecture that supported the development of four uses cases.

**Results:**

Nine participants nodes and 28 Euro-Peristat members completed the deployment of the infrastructure according to the expected outputs. As a consequence, each use case produced and published their own common data model, the analytical pipeline and the corresponding research outputs. All the digital objects were developed and published according to Open Science and FAIR principles.

**Conclusion:**

PHIRI has successfully supported the development of four use cases in a federated manner, overcoming limitations for the reuse of sensitive health data and providing a methodology to achieve interoperability in multiple research nodes.

## Introduction

The extensive and continuous reuse of sensitive health data (e.g. clinical data, electronic health or clinical records, administrative and claims data) could enhance the role of population health research on public decisions.^1^

Nowadays, sensitive health data reuse is still scarce due to data privacy and safety issues; the difficulty to discover data sources of value; complex access rules; uneven data quality; or, limited computational capacities.^2^^,^^3^

Previous European initiatives had shown the difficulties in the reuse and integration of clinical, population and administrative data in population health research. Most of those projects, indicator-based initiatives, used anonymous data pooling after applying harmonization procedures or provided safe remote access to de-identified data or implemented a partial federated approach to data analytics based on the distribution of code.^4–8^ In turn, similar federated approaches to PHIRI’s had a limited scope in the type of research and domain of interest.^9^ All those projects lacked a comprehensive and systematic approach to the interoperable reuse of sensitive data in a federated manner, a methodological approach any type data-driven research, or the proper implementation and deployment of technologies ensuring secure distribution and robust computing. Building on some of these projects, the Joint Action on Health Information (InfAct) implemented and deployed a very small-scale federated network demonstrating how to mobilize sensitive data while complying with the legal and ethical requirements.^10^

PHIRI’s approach can be seen as a federated data network solution.^11^ Federated data networks aim to facilitate accessibility to data without compromising privacy and data governance. These solutions are state-of-the-art for sensitive data sharing; some remarkable examples in the domain are Vantage6,^12^ as an example of computing solution, or the European Health Data Evidence Network (EHDEN),^13^ a project with solutions for data governance and computing.

The Population Health Information Research Infrastructure (PHIRI) has implemented a larger-scale federated multipurpose research infrastructure involving multiple data sources hosted in various European countries. This paper describes the design principles and the different building blocks that have supported the implementation and deployment of PHIRI, the strengths and challenges of the approach and some future developments.

## Methods

In the following paragraphs we formulate the main elements in the design and implementation of PHIRI: (i) the data visiting principle; (ii) the methodological stepwise workflow and (iii) the computational architecture. These elements have been tested in four use cases studying the indirect effects of the COVID-19 pandemic.^14^

### The data visiting principle

The European General Data Protection Regulation (GDPR)^15^ imposes a number of restrictions for the use of sensitive data.

Federated data networks follow the data visiting principle, also referred to as ‘code meets data’ or ‘data centric computing’, to overcome data access restrictions and facilitate research. In this principle, data stays under the control of the owner (data movement is minimized) while users of data (i.e. humans or machine learning algorithms) are allowed to implement federated research solutions.^16^^,^^17^

### PHIRI methodological stepwise workflow

Compliance with the data visiting principle requires a heavy work to ensure organizational, semantical and technical interoperability^18^ that, in PHIRI has been addressed throughout a methodological stepwise approach and a master–worker architecture.

PHIRI stepwise workflow has been consistent with the usual instrumentation of an observational study reusing real-world data; the difference in PHIRI is the need for orchestration of a single research solution across multiple nodes. The PHIRI workflow consists of eight steps.

#### Step 1: Research question

**Figure 1:**
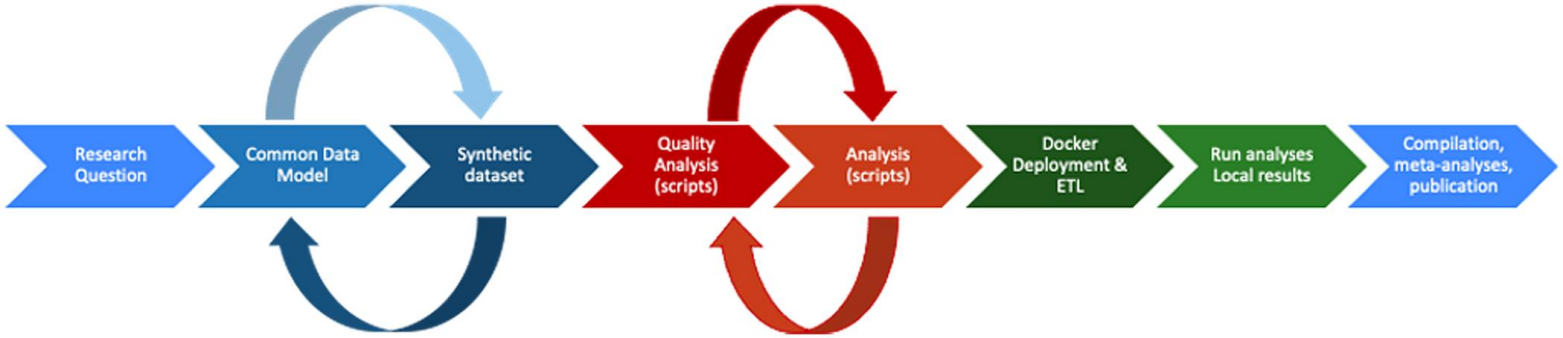
PHIRI methodological stepwise workflow

In figure 1, the stepwise approach starts with the specification of a research question that thoroughly details the cohort of interest; the exposure or intervention of study; the comparator, if any; the outcome or endpoint of study and usually, time of exposure and time to outcome.^19^ This level of specificity facilitates the translation of the research question into the data requirements in step 2.

#### Step 2: Building a common data model

In PHIRI, once the promoter node (i.e. who is leading an inquiry or research project) proposed the research question, a discussion within the federation of nodes took place to translate the question into a common data model (CDM)—a formal document containing the definition of the cohort, the entities and variables of interest, their syntactic and semantic encoding, the definition rules and mappings between controlled vocabularies. After a first version of the CDM was agreed, a recursive process started to determine, according to participants compliance with the CDM, which was the minimum required information that all the nodes were able to accurately facilitate to respond to the research question.

#### Step 3: Producing synthetic datasets

The CDM provides the basis for the construction of synthetic datasets that will allow the implementation and testing of quality assessment scripts and analytical algorithms. PHIRI opted for the use of a parametric approach to synthetic data, generally resorting to non-informative distributions and generated using the ‘Python library faker’.^20^

#### Step 4: Developing the analytical pipeline

The analytical pipeline contains data quality scripts and analytical algorithms. The data quality scripts allowed researchers performing data quality assessments at variable level including elements such as cardinality, missingness, value distributions, and outlier or implausible values. Building on the synthetic dataset, quality scripts were implemented in ‘Python’s ydata-profiling’ library.^21^ In turn, depending on the use case, analytical algorithms yielded graphical representation of monthly counts or standardized rates, empirical break-point analyses, time series forecasting or several generalized linear mixed and generalized additive mixed models.

#### Step 5: Containerization of the pipeline

The analytical pipeline was containerized to facilitate technical interoperability and software maintenance once the pipeline was sufficiently mature. All the documentation (i.e. CDM, synthetic dataset, etc.) and the analytical pipeline, including software dependencies, the database management system and a graphical user interface (GUI) using Vue.js, were locked in a Docker image.^22^

#### Step 6: Distribution of the container image

For distribution purposes, the coordination node distributed the GUI that facilitated a description of each use case, the specific CDM and analytical pipeline, and a series of functionalities including the instructions for local deployment. A demonstrator of the PHIRI App can be found at https://phiri.iacs.es/ and is available in Zenodo.^23^

#### Step 7: Analyses unfold

Once the app was deployed on premise, participants uploaded their datasets, previously transformed according to the specifications of the CDM in step 2, and launched the data quality assessments and the algorithms of analysis. As a result, each participant node achieved the local outputs; thus, tables with aggregated results and interactive graphs (html files) exhibiting the data quality assessments and the research results.

#### Step 8: Transfer of the research outputs

Finally, the local results were returned to the coordinating and/or the promoter node for further joint and comparative analyses and eventually, publication in Open Science FAIR compliant repositories (e.g. Zenodo, GitHub) or in semi-automated reports implemented in QUARTO.^24^

### PHIRI federated computational architecture

**Figure 2:**
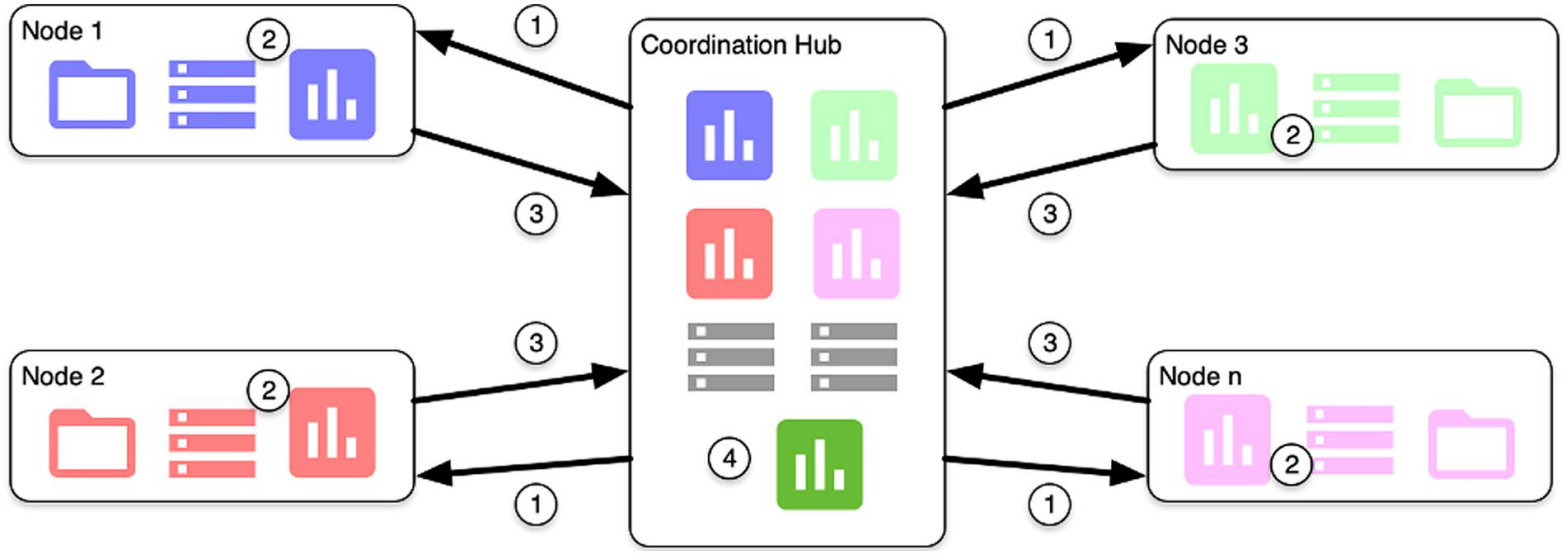
PHIRI computational master–worker architecture

This workflow was supported by a computational architecture that is a network with a ‘master–worker’ topology (figure 2). Thus, (i) the promoter node specified the research query materialized in a CDM with the help of the coordination node; (ii) the coordination node prepared the analytical pipeline and distributed the container images to the participant nodes (step 1 in figure 2); (iii) the participant nodes downloaded the container images and ran the analytical pipeline (step 2 in figure 2) and returned the results to the coordinator node (step 3 in figure 2); and (iv) the promoter node, with the help of the coordination node, compiled the ‘local results’, providing the opportunity for joint and comparative analyses (step 4 in figure 2).

## Results

The PHIRI approach showed to be successful in the deployment of the four use cases. Nine participant nodes and 28 Euro-Peristat members (https://www.europeristat.com/index.php) completed the exercise yielding the expected outputs.

### Use case on the differential exposure of vulnerable population to health care

The research question was: Has the COVID19 pandemic changed patterns of non-COVID19 health care utilization in vulnerable populations within and between countries?

The study comprised different cohorts of patients [i.e. heart attack, stroke, hip and knee replacement and serious trauma admissions from five participant nodes: GöG (Austria), NIPH (Croatia), THL (Finland), University of Swansea (Wales, UK), and IACS (Aragon, Spain)].

The resulting CDM can be downloaded from https://doi.org/10.5281/zenodo.5148013 (v3.0.0) and the analytical pipeline can be downloaded from https://doi.org/10.5281/zenodo.6359850 (v1.1.2).

### Use case on delayed care in breast cancer patients

The research question was: Was there any delay in the first treatment after breast cancer diagnosis associated with the COVID19 first wave stringency measures?

Four participant nodes participated; thus, University of Swansea (Wales, UK), IACS (Aragon, Spain), Cancer Registry (Belgium) and Polytechnic University in Marche (Italy).

The resulting CDM can be downloaded from https://doi.org/10.5281/zenodo.5148021 (v2.0.0) and the analytical pipeline from https://doi.org/10.5281/zenodo.6359892 (v4.0.1). The preliminary outputs can be found at https://doi.org/10.5281/zenodo.6724454. Final results were published at https://phiri.iacs.es/report.

### Use case on changes in perinatal health indicators

The research question was: Were stillbirth and preterm birth rates affected by the pandemic lockdown?

The use case was unfolded in 28 different countries, all members of Euro-Peristat. The implementation of the pipeline focused on developing the CDM and deploying the analytical workflow that, unlike the other use cases, was distributed as separate pieces of code. The resulting CDM can be downloaded from https://doi.org/10.5281/zenodo.5148031 (v2.0.1) and the analytical pipeline from https://doi.org/10.5281/zenodo.6380733 (v2.0.1).

### Use case on effects on mental health services utilization

The research question was: Have hospital admissions, emergency visits and primary care contacts in depression and anxiety endured a change-trend in the aftermath of the pandemic onset?

Seven institutions deployed this use case: GöG (Austria), NIPH (Croatia), THL (Finland), University of Swansea (Wales, UK), IACS (Aragon, Spain), Ministry of Health (Latvia) and the NIHD in Estonia.

The resulting CDM can be downloaded from https://doi.org/10.5281/zenodo.5148039 (v2.0.0) and the analytical pipeline from https://doi.org/10.5281/zenodo.6359904 (v1.1.1).

## Discussion

The following sections discuss the most important aspects of the successful implementation of PHIRI and table 1 provides additional insight on the rationale of our decisions, pros and cons, and contrast to alternatives use in other approaches of the literature.

**Table 1:** PHIRI: appraisal of main implementation elements

Implementation element	Decision	Pros	Cons	Alternatives
Privacy and security by design	Follow the data visiting principle	Minimizing data motion reduces data exposition to breaches.	Data processing becomes more difficult as it requires the development of distributed/federated algorithms	Data pooling with secure processing environments; this approach requires cross-country agreements for data transfer and heavy investments
Semantic interoperability	Use bespoke fit-for-purpose CDMs	Better reflects the study requires and addresses data minimization principle. Contained effort for data holders	Limited scalability when multiple studies with different data requirement run in parallel	*A priori* transformation of data holders’ datasets to a ‘general purpose’ CDM (e.g. Observational Medical Outcomes Partnership (OMOP) CDM) as in the EHDEN network; however, this approach entails heavy data holder investments and may not be fully suitable for numerous research questions.
Data quality	Fit-for purpose data quality assessment embedded in the analytical pipeline on final dataset	Provides participant detailed information on the potential impact of data quality issues in the research results and interpretation	Implies the inclusion of an additional workflow and may entail some additional decision loops.	Fit-for-use: general quality assurance procedures of the main data sources not driven by the purpose of the study; this approach requires the implication of data holders.
Technical interoperability	Use of open-source solutions	No licence fees. Availability of high-end tools supported with large communities. Transparency and reproducibility.	No guarantee of quick support when tools fail. Limited reproducibility	Privative solutions that need contract support. This approach requires heavy investment
	Follow literate statistical programming principle and package in software containers	Reproducibility is enhanced due to simple environment replication and neat and well-documented codes	Increase the programming efforts to produce high quality analysis code and containerization code	Rely on generative AIs to create analysis codes (ChatGPT, Code CoPilot). Use common installers. This approach entails higher supervision costs
	Deploy user-friendly app (assisted by a graphical user interface) to launch the local analyses	Ease the interaction with main features and functionalities for the federation solutions	Increase the development costs	Basic command line solutions (e.g. basic Python or R scripts). This approach makes complex software versioning maintenance and technical interoperability
	Follow FAIR principles for resulting digital objects	Maximize the reuse of the project outcomes	Including an additional workflow	Disseminate the results in common channels (e.g. only scientific papers); this approach implies reducing the reusability of the digital objects
Organizational interoperability	Decoupling tasks as participant nodes from tasks as data holders	Reduces the risk for data holders; only focus on granting access to data	Adds an extra layer of governance increasing the overheads on the research process.	Just data holders can be participant nodes; this approach impedes providing context and domain expertise to data
	Choosing asynchronous analysis as the default approach	Keeping nodes pursuing the deployment of the use case irrespectively of delays in data access	Federated learning approaches relying on synchronous analyses are more complex to implement	Postpone analyses until all participant nodes have the data available or limit participation to data holders; this approach put at stakes commitment of the front-runners
Technical capabilities	Building technical capabilities in the research consortium based on strong documentation and helpdesk hands-on training	Free availability of documentation of all the digital pipeline plus IT experts’ assistance enables significant learning.	Relies on the existence of IT experts in the participant nodes. Need for the curation of all the documentation produced in the federation.	Privative solutions with continuous professional support. Use consulting firms to provide professional support. This approach entails heavy investments in licences and consulting/technical support services

### Privacy and security by design

A major difficulty in the extensive reuse of sensitive data in population health research are data holders’ concerns on data protection and security.

PHIRI has adopted a methodology that limits extensively the exposure of individual level data to malicious actors procuring a number of safeguards. Firstly, the data visiting principle where only code and results move and sensitive data remains under the jurisdiction of data holders, where their access, security and disclosure procedures prevail. Secondly, code moves contained in a standalone solution that can be run isolated from the systems and it is auditable for security checks. Finally, the data access application is limited to those variables required in the CDM, thus complying with the data minimization principle.

### Semantic interoperability

Another major difficulty when reusing real-world data is the poor standardization, particularly, when reusing data from different data sources in several countries. As data holders do not have their collections fully standardized nor use semantic standards common to all data collections in all data hubs, and, not all the attributes of interest in a research project have a semantic standard of application, there is a need for the implementation of a layer of semantic interoperability. The PHIRI fit-for-purpose CDMs is a simple yet useful way to quickly tackle semantic interoperability.

### Data quality

In the reuse of data for observational research, researchers have to assume a certain degree of inaccuracy in the data. This inaccuracy may (or may not) affect the robustness of research. PHIRI has implemented a formal procedure to assess quality including several quality checks at variable level applied to the specific dataset of the project. All the participant nodes in the federation perform the same assessment providing the opportunity to understand potential differences in the results that come from data quality issues.

### Technical interoperability

PHIRI has been built on a number of technical principles. Firstly, the selection of open-source technological solutions allowing software development transparency, and easing code persistence and traceability. Secondly, the implementation of the analytical pipeline has followed the literate statistical programming approach enhancing reproducibility. Thirdly, usability in the execution of the analyses throughout a GUI. Fourthly, designing PHIRI to allow any federated research, including federated learning; and finally, making all the digital objects FAIR. A critical appraisal of the technical solutions are available as supplementary materials (Supplementary table S1).

### Organizational interoperability

Apart from a governance build upon strict compliance with the stepwise workflow, the different nature of the PHIRI nodes (health authorities, public health bodies, data holders and research and academic institutions), some of them also data holders, required decoupling their capacity as participant nodes from their responsibilities as data holders. Decoupling has limited the risk for those nodes also acting as data holders to only focus on granting access to data permits.

An important decision for decoupling was implementing an asynchronous approach to the resolution of the research queries. There was no explicit requirement for participants to have immediate access to the data; on the contrary, the design allowed each partner to contribute data and run their local analysis when they had access granted and contribute to the joint analyses later in time. A web application was developed to facilitate the semi-automation of the collection of local outputs and the production of joint analyses when a ‘late’ dataset is ready.^25^

### Technical capabilities

A federated approach relies on IT capabilities across nodes in the federation. However, IT capabilities within the participant nodes with regard to IT tools were found uneven and, in some cases, limited both, in terms of systems administration (ETL process, Containers deployment or Linux AdminSys) and analytical support (use of SQL queries, R or Python). Notably, within those participant nodes, slightly more than half (53.8%) declared to have limited capabilities in the deployment of Docker images, and 38.5% recognized the need for support in general IT administration and data analytics. Despite the use of the GUI, there was a need for the creation of a help desk that guided the participant nodes in many instances of the process.

### Future developments

For PHIRI to be an infrastructure of research services, there is a need for migrating from a master–worker architecture with strong human dependencies towards a peer-to-peer network where processes are designed to foster machine-to-machine interaction. Moreover, PHIRI should consider a new landscape where the European Health Data Space for secondary use (HealthData@EU) and the European Open Science Cloud (EOSC) will likely be providing some services for research communities.

### Towards a peer-to-peer network

The current PHIRI master–worker architecture has relied strongly on the coordination node who has covered any difficulty in the deployment of the four use cases. Should PHIRI want to cover many more research questions allowing more participant nodes to contribute, there is a need for a change in the architecture, moving towards a peer-to-peer network, where every single node may dynamically act as coordinator, promoter or participant.

### Towards a machine-to-machine interaction

In parallel to the transition to a peer-to-peer network, it will be also important to automate as many activities as possible. The current human-to-machine approach in PHIRI should be upgraded with a machine-to-machine (m2m) protocol exchange. In a m2m scenario, the analytical pipeline executed in the participant nodes would automatically push the local results producing joint analyses. This communication will require the inclusion of other technical components, such as an authentication, authorization and identification protocols or secure application programming interfaces, not in the scope of PHIRI.

**Figure 3:**
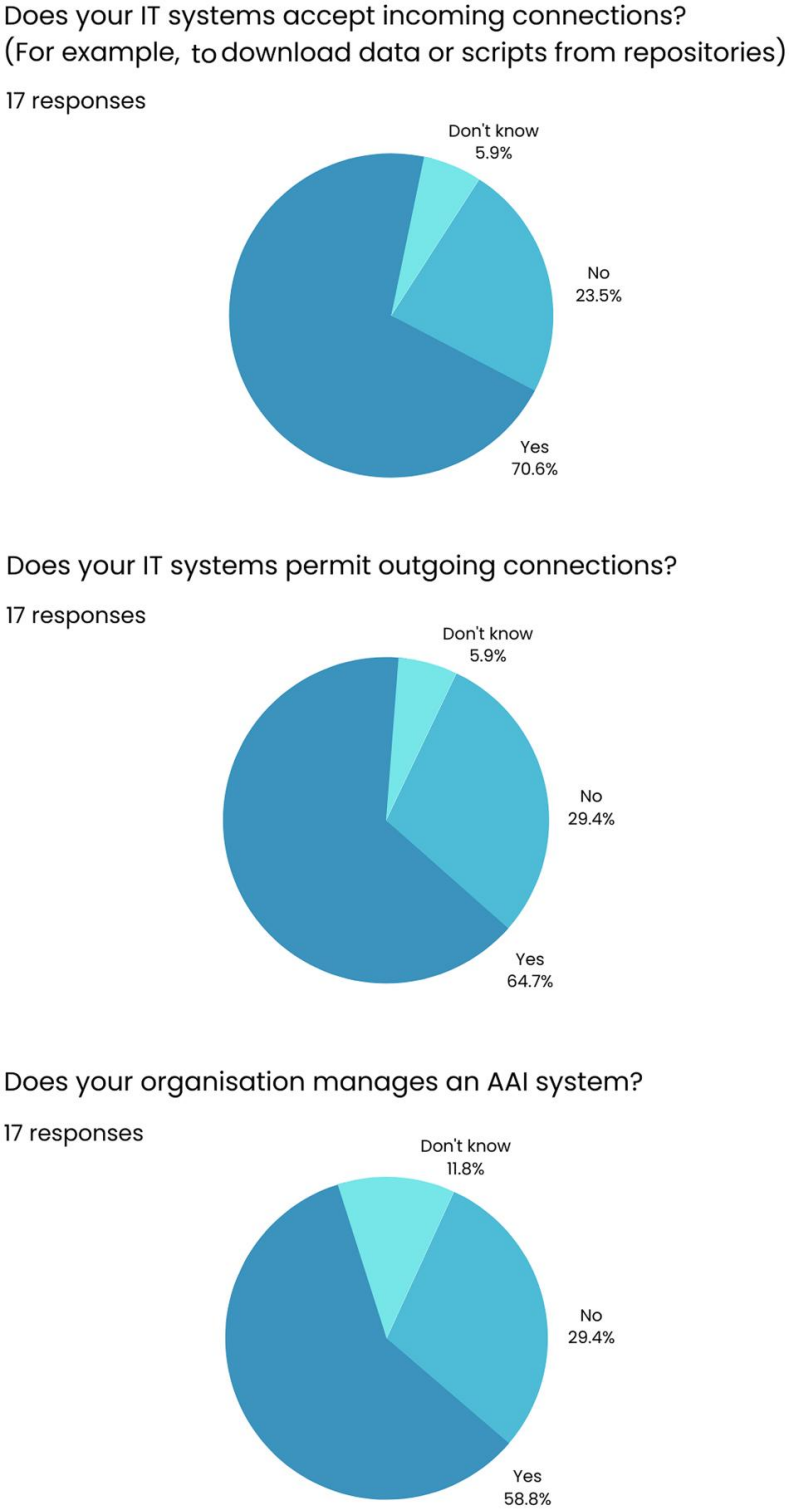
Preparedness for a PHIRI m2m approach

A scoping survey to participants (figure 3) provided a notion of how feasible moving towards an m2m approach could be. Out of 16 institutions participating in the use cases, 62.5% managed AAI systems and allowed incoming connections, with 56% also allowing outgoing connections.

### Integration in the EU health data reuse landscape

PHIRI has been unfolding and adapting consistently to the incipient roadmap of HealthData@EU^26^ and to the developments of EOSC. With the caveat that HealthData@EU services and architecture are still under discussion, PHIRI has been thought to be fully interoperable; thus, at organizational level since participant nodes in PHIRI will be actually acting as data holders or health data access bodies in the HealthData@EU; at semantic level since PHIRI CDM fit-for-purpose approach is compatible with any future development on semantic interoperability at larger scale; and, at technical level since PHIRI technical infrastructure has been thought to be reused as a technology within the foreseeable deployment of Secure Processing Environments in HealthData@EU.^27^

When it comes to the EOSC landscape, PHIRI has been one of the early adopters in the EGI-ACE project (Advanced Computing for EOSC)^28^ PHIRI may take advantage of some of the computational EGI services such as the development of playgrounds with synthetic data resembling datasets in available at HealthData@EU.

## Conclusion

The PHIRI federated approach builds on the data visiting principle, ensuring privacy and security by design. By minimizing data movement and keeping data in its original location, the infrastructure aims to reduce privacy risks associated with unnecessary data transfers.

The infrastructure has been designed following the current trends on federated data networks to successfully govern legal, organizational, semantic and technical interoperability when reusing sensitive data from many data sources and holders and has deployed a workflow methodology that has been proven successful in the orchestration of four research projects.

The evolution of PHIRI should enable its integration in the new data reuse European landscape, whose main technical pillars should be the deployment of a peer-to-peer computational network and a machine-to-machine approach to federated research.

## Supplementary Material

ckae036_Supplementary_Data

## Data Availability

This article describes the technological development of PHIRI. Not applicable. Key points PHIRI has successfully supported the development of four use cases in a federated manner, providing a methodology to achieve interoperability in multiple research nodes accessing a variety of data sources. By minimizing data movement and keeping data in its original location, the infrastructure has effectively overcome the limitations for the reuse of sensitive health data while complying with legal and ethical provision on the matter. Large scale developments of PHIRI will depend on progressively transforming the current ‘master–worker’ architecture in a peer-to-peer network where nodes are able to assume co-leadership and methodological and technological roles.
