# Supplementary material for: PHIRI: lessons for an extensive reuse of sensitive data in federated health research
Source: Eur J Public Health. 2024 Jul 1;34(Suppl 1):i43–9. doi: 10.1093/eurpub/ckae036 (PMC11215320; doi:10.1093/eurpub/ckae036)
Supplement: ckae036_Supplementary_Data [file ckae036_supplementary_data.docx]

**Supplemental material**

**Table S1. Analysis of the technical solutions used in the PHIRI analytical workflow.**

| **Solution** | **URL** | **Rationale** | **Pros** | **Cons** | **Alternatives** |
| --- | --- | --- | --- | --- | --- |
| Python Faker | <https://faker.readthedocs.io/en/master/>  <https://github.com/joke2k/faker> | Most used Python library for synthetic data generation (~350K daily downloads) | Wide number of data types available. Easy to generate other types of data. Capacity to tune localised data generation (addresses, telephone number formats) | Not all localisations are available for all data types / fields | Python: Trumania, Synthetic Data Vault,  Other: synthpop (R), fabricat (R) |
| ydata-profiling | <https://ydata-profiling.ydata.ai/>  <https://github.com/ydataai/ydata-profiling> | Most used Python library for profiling (~25K daily downloads) | Simple interface to produce a detailed number of quality metrics. Sensitive data handling features. Heavy development of Ydata.ai company. | Univariate exploratory data analysis readily available but requires expert-level python knowledge for data quality assessment customisation. In addition, it may create processing bottlenecks when handling large volumes of data. | Python: data-quality-check, data.validator, Dora, pydqc  R: dlookr, data.validator, DataExplorer |
| Vue.js | <https://vuejs.org/> | Highly used. Capacity | Complete framework for building web user interfaces.  Easy to use and customise, with good performance and versatility. | Heavy data and processing volumes. Dependent on Node.js and NPM | Python: Svelte, Streamlit  R: Shiny; Quarto |
| Docker | [https://www.docker.com](https://www.docker.com/) | Industrial standard for software container solution. Large community | High number of existing images to compose and to build. | Operates at root level may prevent its deployment in some settings | Singularity, Podman |
| QUARTO | <https://quarto.org/> | Evolution of R Markdown to more flexible solution including multi language support | Solid evolution of a de-facto standard data science for reporting | Multiple dependencies both in R and Python | R: rmarkdown  Python: Jupyter-book |
